# Opportunities and challenges for the introduction of a new female condom among young adults in urban Zambia

**DOI:** 10.1186/s12978-019-0839-x

**Published:** 2019-12-03

**Authors:** Katherine Gambir, Jessie Pinchoff, Olasubomi Obadeyi, Thoai D. Ngo

**Affiliations:** 1 Independent Consultant, One Dag Hammarskjöld Plaza, New York, NY 10017 USA; 2 Poverty, Gender, and Youth Program, Population Council, New York, NY 10017 USA; 3 Innovations for Poverty Action – Zambia Office, Plot 26, Mwambula Street, Jesmondine, Lusaka, Zambia

**Keywords:** Female condom, Zambia, Young adults, Adolescents, Urban, Contraception, Reproductive health, Family planning

## Abstract

**Background:**

Expanding contraceptive method choices for sexually active youth is critical to prevent STIs/HIV and unintended pregnancies. However, preferences and decision making around contraception among young adults are not well understood. A new female condom (FC), the Woman’s Condom (WC), features an improved design and is marketed as a premium product at a higher price point. We conducted a qualitative study to examine the underlying knowledge, attitudes, and perceptions around the FC generally, the WC specifically, and to explore the opportunities and challenges of introducing the WC to young adults in urban Zambia.

**Methods:**

Thirty focus group discussions comprised of 245 men and women aged 18–24 years were facilitated by local moderators in Lusaka, Zambia between August and December 2016. Data were analyzed using thematic content analysis using ATLAS.ti (Version 7.5.17).

**Results:**

While interest in the newly launched WC was high, few participants recognized it, so discussion was framed around FCs more generally. The decision to use a FC is challenged by misconceptions regarding safety and correct use, cost, and women’s limited power over decision making in relationships. Participants also reported low availability of the product. Several opportunities for FC uptake were discussed, including the perception that FCs provide better sensation during intercourse compared with male condoms, and women reported enthusiasm for the opportunity to be the partner responsible for contraception. Some women expressed interest in the WC in order to ensure it was not tampered with by their partner, a practice commonly reported by both genders that reflects high levels of mistrust.

**Conclusions:**

Findings suggest the FC, including the new WC, has potential to increase gender equity by giving women a product they are comfortable buying and being able to control to ensure correct use; however, future programming should address high perceived cost of the WC and women’s limited decision making power in relationships. Findings also highlight the need for interventions that include product demonstration and promote the WC as a method that provides better sensation during sex than male condoms. To increase voluntary uptake, the challenges identified here should be incorporated into future social marketing campaigns.

## Plain English summary

Young, sexually active people need more contraceptive options to protect themselves from HIV, sexually transmitted infections, and unintended pregnancies. We interviewed groups of young people to learn about their preferences, and whether they would use a new type of female condom (FC), called the Woman’s Condom.

We aimed to find out what urban youth know and think about the FC, and the Woman’s Condom specifically. We conducted 30 group discussions including 245 men and women between the ages of 18 and 24 years in Lusaka, Zambia. We reviewed the discussions, developed important topic areas that were discussed, and noted what was similar and different about the responses.

We learned that not many young adults knew about the Woman’s Condom, so the discussions were mainly around FCs in general. Young people said they do not use the FC because they did not know how to use it correctly, and had concerns regarding its safety and effectiveness. However, young people were also excited to try the Woman’s Condom, because they thought it would feel better during sex compared with a male condom, and some women expressed excitement to be the ones responsible for using the product.

Our research identified some potential barriers to the uptake of the Woman’s Condom. However, these may be possible to overcome given the enthusiasm expressed by participants for a new product controlled by the woman. Marketing campaigns that can address concerns and promote the Woman’s Condom may increase overall contraceptive use among urban young adults.

## Background

Globally, 214 million women experience an unmet need for family planning (FP) [1], with over 100 million citing method-related reasons for non-use of modern contraceptives [2]. Renewed global attention and action are being directed toward developing the female condom (FC) market and ensuring that products are widely available [3]. Global distribution of FCs more than tripled from 2005 (13.5 million) to 2009 (50 million) [4]. The FC is a barrier method of contraception inserted into the vagina that protects against both HIV/STIs and unintended pregnancy making it the only dually-protected and female-initiated method.

The FC products available, such as the FC2, have similar designs, but varied materials. These FC products have encountered several barriers to uptake, such as an unappealing design and low availability [5–7]. To date, FCs only represent 0.8% of the total condoms distributed by donor nations [8]. While several countries that actively promoted FC use, such as South Africa, Brazil, Ghana, and Zimbabwe, have seen increasing sales, uptake in other countries has been lower than anticipated [9]. For example, in Cameroon, despite relatively high awareness of the FC among a sample of men (74.5%) and women (88.5%), only 8 and 9.9% respectively reported having ever used them [10]. In response, a third generation FC product, the Woman’s Condom (WC), has been developed by the Program for Appropriate Technology in Health (PATH) [7]. The WC has an improved design, featuring a thin, flexible pouch made of polyurethane film that conforms to the shape of the vagina and a flexible, soft outer ring that protects the external genitalia. The WC’s unique design offers easy insertion and removal, a secure fit, and good sensation during sex [11]. Formative studies show that the WC has lower rates of clinical failure and is associated with fewer adverse events than the FC2 [12]. A multi-site acceptability study suggests the WC is preferred over the FC2 by users [7, 12].

Zambia is an important context for the WC because of its high unmet need for FP (27.1%) and high HIV incidence (13.3%) [13]. Young women aged 18–24 years, particularly in urban areas, bear a disproportionate burden of the HIV epidemic [9]. However, use of modern contraceptives remains moderate; about 45% of women report using any modern method, and less than 1% report using a FC [13]. Expanding contraceptive choice for sexually active young adults is critical, especially as they are entering their most productive years through workforce engagement and advanced schooling.

Zambia’s first FC program was supported by USAID beginning in 1992 in response to the global AIDS epidemic. Evidence on the program’s impact is limited. However, research on the uptake of FCs in Zambia is growing; one study suggested that the FC was likely to be most important for males and females ages 15–49 years in Zambia who were unable or unwilling to use a male condom [14]. In 2004, USAID-PEPFAR funds supported the Expanding Effective Contraceptive Options (EECO) project, which was designed to support the research, development, and introduction of new contraceptive methods that address method-related reasons for non-use, expand contraceptive choice, and increase access to woman-initiated methods to better meet the reproductive health needs of women and girls [11]. To better address the reproductive health needs of women and girls in Zambia, the EECO project launched a national distribution of the WC under the brand name *Maximum Diva Woman’s Condom*, coupled with a social marketing campaign in 2016. Society for Family Health (SFH), the Zambian subsidiary of Population Services International (PSI), distributed the WC through pharmacies, private health facilities, and other outlets (e.g., bars, chain stores, container stores, convenient stores, drug stores, groceries, hair salons) in 40 urban wards in and surrounding Zambia’s capital city, Lusaka. The introduction of the WC to the public and private sectors was coupled with a marketing and interpersonal communication (IPC) outreach campaign targeting young men and women (18–24 years) in the distribution area .

FCs offer dual protection and are female initiated; the WC is the newest iteration of the FC product made available for the first time in Zambia. The recommended price for the WC in Zambia is K10, or USD$1 compared to the male condom which is marketed for USD$0.50 for three condoms and are freely available in many public health facilities [15]. The WC is being marketed as a premium product to young adults in urban areas.

Studies suggest that expanding contraceptive options increases the likelihood of use of any method [16]. The WC may fill a gap in contraceptive options; however, introduction alone will not increase uptake, and the perceptions of FCs among a general young population has not been well studied. This qualitative study builds on previous work under EECO to understand the context of FCs in urban Zambia [17], and aims to investigate youths’ perceptions around female-initiated contraceptive methods to understand the opportunities and challenges for uptake of the WC among a young, urban population in Zambia.

## Methods

This qualitative study was nested within a broader evaluation designed to assess the impact of the distribution, mass media campaign, and a randomized interpersonal communication intervention on the uptake of the new WC. The evaluation is described in the study protocol [18]. To examine the underlying meanings of youth’s knowledge, attitudes, and perceptions around the FC generally, and the WC specifically, a set of qualitative focus group discussions (FGDs) were conducted. A total of 30 FGDs were conducted with young people (18–24 years old) in Lusaka, Zambia; of these FGDs, 9 were men only, 9 were women only, and 12 were mixed sexes.

This study received ethical approvals from the Institutional Review Board at Innovations for Poverty Action (IPA) and the Zambian Excellence in Research Ethics and Science, as well as written permission from the Zambian Ministry of Health. All information collected in the FGDs has been de-identified to ensure confidentiality. Each participant signed an informed consent form.

Participants aged 18–24 years living in the 40 wards comprising the study area were purposively sampled. Participants reported variation in marital status and childbearing, representing mixed perspectives and experiences of urban young adults. To recruit participants, a 500-m radius was drawn around a central location (usually a marketplace) in each ward. Participants in all of the intervention wards (*n* = 20) and half of the control wards (*n* = 10) were recruited within the 500-m radius, and in the other half recruited from outside the 500-m radius (*n* = 15). A total of 245 individuals were included in the FGDs comprised of 114 women and 131 men.

Data were collected between August 24 and December 20, 2016, beginning approximately 4 months after the start of the intervention and WC distribution. Open-ended, semi-structured questionnaire guides were used to explore various domains regarding modern contraceptives, specifically the FC. Domains included general contraceptive knowledge, general condom knowledge, WC knowledge, and couples’ communication around condom negotiation. The discussions were facilitated in the local language, Nyanja, and in English. FGDs were held in private areas (e.g., hotel conference space) to ensure privacy and confidentiality. FGDs were led by a trained moderator and notetaker, and were audio recorded. The moderators were young Zambian adults (ages 18–30). A young man moderated the male only groups and two young women moderated the female and mixed gender groups. No personal identifying information was obtained.

Two in-house transcribers were trained and supervised by the local research team. Members of the local research team reviewed a random sample of transcripts. If significant differences between the transcriber’s work and the original transcript existed, then the complete transcript was reviewed by the research team and transcriber and reconciled by consensus. Analysis of the data was conducted by the lead study authors with support from the local research team using thematic content analysis, whereby transcripts were reviewed several times to create a code book with categories and key words selected based on reoccurring themes identified in the transcripts. These codes were subsequently analyzed within and across discussions to determine trends, patterns, frequency, and relationships among the codes using ATLAS.ti (Version 7.5.17) by which the themes were further explored and refined.

## Results

This section characterizes the challenges and opportunities in introducing the WC in urban Zambia as reported by FGD participants. Because the WC is relatively new, most participants were not familiar with it. Therefore, discussions centered around FCs more generally, but where the WC was specifically mentioned, this acronym is specified.

### Challenges

#### Safety concerns & misconceptions

Both male and female participants expressed similar concerns regarding FC use, reflecting low knowledge of correct insertion, safety, and use. For example, participants reported the misconception that because FCs have two “rings”, they areopen on both sides, and sperm can pass through (The WC does not have this feature). This led to confusion regarding the FC’s efficacy. Participants also expressed concern that they, or their partners, could be physically harmed by using the FC. One man explained his fear that the FC could remain stuck inside the woman’s body:

*“Because the female one you wouldn’t know; you may be surprised that as you make love, all of it is pushed inside. It may break inside and you will be shocked. Some females will feel shy to go to the hospital when it's stuck inside her. She will be shy to mention it at home and meanwhile it is getting to a week. She begins to have infections.”* (Man, Male Only FGD)

Another common misconception among participantswas that the FC had to be inserted many hours before sex. Many young adults reported that they preferred to use the male condom because sex can occur immediately after application, but they were not sure this was true for a FC. One man in a male only FGD reported that a couple must wait 24 h after FC insertion to have sex. This confusion may stem from previous messaging around FCs. The FC2 was marketed with the message that it could be inserted up to 8 h before sex, and the WC 5–15 min before sex. However, these insertion times are not required. Several participants reported confusion about this wait time leading to decisions not to use the WC.

#### Cost

Cost was another concern. In launching the WC, a more premium product with a higher price point compared to male condoms was placed into the market. Some participants expressed concern that the higher price may limit the ability of young adults to purchase them. One woman noted the importance of ensuring the WC was affordable:

*“They are people who are less privileged, so they won’t buy. We have people suffering that 6 kwacha they don’t see in months so I think if it’s too expensive people won’t buy.”* (Woman, Female Only FGD)

Some women reported not using FCs because they were not available on the market. When asked by the facilitator why FCs are not common, one woman responded:

*“I heard that they enter the uterus when having sex, so they started discouraging shop owners from selling them. They are not common. You can hardly find them.”* (Female, Female Only FGD)

In comparison, male condoms are free and widely available at most clinics in Lusaka, where young women reported procuring them most frequently. They are also available at very low prices in shops and grocery stores, where young men reported that they were more likely to procure them.

#### Gender Norms

Gender norms around who is responsible for purchasing specific types of contraception was reported as a barrier to uptake. Typically, both genders agreed that within a sexual partnership, men are responsible for purchasing condoms. Men reported a few barriers to purchasing male condoms (e.g., fear their family would find out about the purchase); however, they were even less willing to buy FCs because this is a product "for" women.

Young women are not often responsible for purchasing condoms and may not feel comfortable going to a shop to make this purchase. One concern cited is that women seen purchasing condoms may be labeled as promiscuous. One woman explained:

*“They can think otherwise of you that, “Why is this female often buying condoms?” They will think you are immoral. It is better for the males to buy and then they put it in the pocket.”* (Woman, Mixed FGD)

Overall, the major challenges to uptake of the WC were misconceptions regarding FC safety and use, cost, and gender norms around purchasing gender-specific condoms.

### Opportunities

Some participants, mostly women, reported interest in and willingness to try the FC, representing potential opportunities for its promotion and use. Further, those who had experience using a FC, or who learned about the WC from other participants during the FGDs, expressed willingness to use it in the future. Below are themes that emerged as potential opportunities for the WC.

#### Mistrust

Mistrust in sexual relationships was a frequent theme among the FGDs, and a key reason some women reported a potential preference for a FC compared to a male condom. Both male and female participants believed sexual partners may tamper with the male condom. Women feared that men may remove the male condom during sex and both men and women believed their partners may prick holes in the male condom to cause pregnancy or transmit STIs, including HIV. As a result, some women suggested the FC may be preferable to the male condom as it would allow them to control their protection during intercourse. One woman explained:

*“Since you do not trust him. He might cut the tip of the male condom, but if it is you putting it on, you are assured of safety*.” (Woman, Female Only FGD)

The FC would potentially allow women to exert control over the decision to use contraception and provide assurance that it is being properly used.

#### Sensation

Many men (and some women) were interested in trying the WC because they do not like the feeling of having sex with a male condom. One female explained:

*“Most guys say you can’t eat a sweet with its cover, you will need to remove it in order to enjoy it. They would like to have sex without a condom. Even girls say so.*” (Woman, Female Only FGD)

The above quote is an example of the idiom many participants used to explain why youth do not use male condoms. Men were eager to determine whether sex with a FC would be more enjoyable than with the male condom. One male explained:

*“I can use it because I would love to experience how it feels to use. That is why I would want to use it. To determine how important it is and see if I feel the same as I do when I use a male condom or when she wears the condom.”* (Man, Mixed FGD)

#### Women taking control

Given many men prefer not to use a male condom and women often lack the power to negotiate male condom use, some women reported an interest in being the party responsible for using a condom. One woman explained:

*“Because it’s amazing someone has come up with something like them and it gives us the advantage that even if the guy doesn’t want to use the condom, we now can take up that responsibility*.” (Woman, Female Only FGD)

Although most women were not comfortable buying male condoms, some reported they would potentially be more comfortable buying the WC. Women cited confidence in having control over condom use as a motivating factor for why they would buy the WC.

Overall, men were more comfortable buying male condoms compared to women; however, some men were not comfortable buying FCs. One man explained:

*“Well, I am a man and I have to buy a male condom. When it comes to female condom, I don’t think I can go buy a female condom because I am a guy. I can’t use it. It doesn’t make sense.”* (Man, Mixed FGD)

This quote represents the sentiment of many young men that believe it is their responsibility to buy male condoms, but not FCs, because these are used by women. In this case, they believe women should be responsible for buying female-initiated contraceptives such as the FC.

Overall, most women expressed willingness, and even excitement, in the idea of being responsible for contraception, and some men supported the idea of women purchasing FCs.

## Discussion

The new WC product faces several challenges and potential opportunities as it is being introduced into the contraceptive mix in urban Zambia. The challenges are mainly related to safety concerns and misconceptions regarding correct use of this type of condom. Despite these challenges, FGD participants expressed interest in trying the FC citing the perception of improved sexual sensation, and the opportunity for women to take on the responsibility of procuring and using the condom. Other positive perceptions were associated with addressing the mistrust reported between sexual partners, with the potential for the WC to offer women an opportunity to ensure proper use and no tampering with the product by their partner. These discussions offer insight and contextualization into the marketing of the WC and potential opportunities to increase WC uptake among young people in urban Zambia.

Overall, findings from this study are consistent with the literature. Many participants expressed misconceptions about all forms of modern contraception, but misconceptions related to the WC may be straightforward to correct with the dissemination of accurate information and proper training regarding insertion and use. Published studies report that the overall acceptability of the FC improves over time with practice [7, 12, 19]. One key point for clarification that was discussed in the FGDs was that the WC must be inserted for many hours before sex. This misconception may be due to a commonly mentioned advantage of FCs, that “they can be inserted hours ahead of a sexual encounter” [20, 21]. Steps should be taken to clarify that the WC is inserted only 5–15 min prior to intercourse; whereas other FC products can (optionally) be inserted farther in advance of sex. During the FGDs, once these misconceptions were addressed, many participants expressed interest in trying the product.

The WC may fill a critical gap for young women who want to take control of their contraception, but don’t feel comfortable buying male condoms [22, 23], or where they may not be able to negotiate male condom use [8]. For example, condom use failure is often attributed to alcohol and other drug use [24], and linked to intimate partner violence [25]. However, in promoting the WC as a female-initiated contraceptive method it is critical to consider potential negative repercussions of shifting the burden of responsibility for providing condoms to women. First, some studies have shown that female-initiated interventions are counterproductive because men must be involved in contraceptive decision making to be successful [8, 20, 21, 26]. Second, the WC is more expensive than the male condom (USD$1 for two WC vs USD$0.50 for three male condoms), so women may take on a cost burden as well as the responsibility to carry the WC. Female FGD participants expressed mixed reactions to the possibility of buying the WC; some women reported excitement and others were unsure they would feel confident buying the WC.

A topic that came up frequently in the FGDs was mistrust between sexual partners, particularly that the suggestion of condom use could be associated with infidelity. Both men and women expressed distrust for the opposite sex. This concern has been frequently reported as a barrier to male condom use [20, 26, 27]. It is less clear whether these same feelings of mistrust may persist with the WC, but two issues are important to address. First, some studies highlight that this negative association may be related to FC marketing. In some countries FC promotion as a female-controlled method backfired because males viewed FCs as threatening to their control over their partner’s sexual behavior [28, 29]. Second, many FC distributions have targeted FCs toward sub-groups such as sex workers, reinforcing stereotypes. Promoting the FC to a more general population may reposition the product, reduce stigma, and improve acceptability [8].

### Study strengths and limitations

This study has a few limitations. First, the availability of the WC specifically was low overall, as it was not available in many outlets due to the newness of the product in the Zambian market. Therefore, a majority of the participants were not familiar with the WC and reported perceptions or knowledge could have been referring to the WC or other FC products. Due to challenges distinguishing between products, we refer throughout the paper to FCs generally (to include WC and other FC products) regardless of the brand, unless the WC product was named specifically by participants.

Second, study findings are not necessarily generalizable to all young adults in Sub-Saharan Africa. However, given the qualitative results reached saturation, this suggests our findings may reflect the views and perspectives of young adults living in these urban settings.

Third, social desirability bias may have led to altered responses if participants believed the facilitators were employed by organizations promoting contraceptives, such as the WC. Therefore, participants may have reported more favorable perceptions regarding FC than they held to appease the facilitators. To mitigate this potential bias, the moderators did not identify themselves as being affiliated with an organization promoting FCs, or the WC. Also, we hired moderators with similar backgrounds as the study participants to ensure participants were comfortable sharing their views and perspectives during the sessions.

## Conclusions

First, findings suggest FCs, such as the recently marketed WC, have the potential to increase gender equity by giving women a product they are comfortable buying and being able to control to ensure correct use. Second, challenges around condom negotiation may persist even with the WC, so policies and programs should continue to address gender norms and power dynamics around contraceptive use [30, 31]. Third, WC marketing should consider strategies, such as male engagement, to prevent potential backlash among men regarding this female-initiated contraceptive. Finally, behavior change interventions that include WC demonstration have potential to improve youth’s comfort level with FCs and shift attitudes in a positive direction [32].

To promote voluntary use of a new contraceptive method, proactive and well-planned strategies are necessary to integrate them into the available contraceptive method mix [16]. Despite its low availability, urban adolescents and young adults in Lusaka expressed interest in trying a new FC, such as the WC. To increase use within the context of informed choice, policy and programmatic efforts should ensure availability of the product in outlets where women shop, and teach couples how to correctly use FCs, including the WC, correcting misconceptions. Gender equity, condom negotiation skills, and cost are also important to address. Findings from this study suggest future lines of inquiry should include whether WC promotion leads to any unintended consequences, such as shifting the burden of purchasing contraception from men to women.

## Data Availability

To protect the autonomy and confidentiality of respondents, qualitative interview transcripts or audio will not be made available to the public, but are available from the authors upon reasonable request and with permission from IPA.

## Abbreviations

EECO: Expanding Effective Contraceptive Options
FC: Female Condom
FGDs: Focus group discussions
HIV: Human immunodeficiency virus
IPA: Innovations for Poverty Action
IPC: Interpersonal communication
PATH: Program for Appropriate Technology in Health
SF: Society for Family Health
STI: Sexually transmitted infection
WC: Woman’s Condom
